# Fractionation of Stable Cadmium Isotopes in the Cadmium Tolerant *Ricinus communis* and Hyperaccumulator *Solanum nigrum*

**DOI:** 10.1038/srep24309

**Published:** 2016-04-14

**Authors:** Rongfei Wei, Qingjun Guo, Hanjie Wen, Congqiang Liu, Junxing Yang, Marc Peters, Jian Hu, Guangxu Zhu, Hanzhi Zhang, Liyan Tian, Xiaokun Han, Jie Ma, Chuanwei Zhu, Yingxin Wan

**Affiliations:** 1 Institute of Geographic Sciences and Natural Resources Research, Chinese Academy of Sciences, Beijing 100101, China; 2 Institute of Geochemistry, Chinese Academy of Sciences, Guiyang 550002, China; 3 Shenyang Academy of Environmental Science, Shenyang 110016, China; 4 College of Applied Arts and Science of Beijing Union University, Beijing 100191, China

## Abstract

Cadmium (Cd) isotopes provide new insights into Cd uptake, transport and storage mechanisms in plants. Therefore, the present study adopted the Cd-tolerant *Ricinus communis* and Cd-hyperaccumulator *Solanum nigrum*, which were cultured under controlled conditions in a nutrient solution with variable Cd supply, to test the isotopic fractionation of Cd during plant uptake. The Cd isotope compositions of nutrient solutions and organs of the plants were measured by multiple collector inductively coupled plasma mass spectrometry (MC-ICPMS). The mass balance of Cd isotope yields isotope fractionations between plant and Cd source (δ^114/110^Cd_organs-solution_) of −0.70‰ to −0.22‰ in *Ricinus communis and −*0.51‰ to −0.33‰ in *Solanum nigrum*. Moreover, Cd isotope fractionation during Cd transport from stem to leaf differs between the Cd-tolerant and -hyperaccumulator species. Based on these results, the processes (diffusion, adsorption, uptake or complexation), which may induce Cd isotope fractionation in plants, have been discussed. Overall, the present study indicates potential applications of Cd isotopes for investigating plant physiology.

Cadmium (Cd) is a highly toxic heavy metal that can be accumulated in the human body through the food chain^1^,^2^. The health risks of environmental Cd pollution have caused global concern, since the ‘itai-itai’ disease caused by chronic Cd poisoning appeared in Japan in the 1950’s^3^. As a cost-effective and environmentally sustainable strategy^4^, phytoremediation could be used in the remediation and sustainable management of Cd polluted soils^5^. The mechanisms of Cd uptake, transport, and storage in plants are of high interest with respect to phytoremediation of Cd polluted soils.

Metal isotope signatures can be applied to identify the chemical process controlling metal transformation in plants and organisms^6^. Previous researchers have studied the metal toxicity in plants using different concentrations and forms of heavy metals^7^,^8^. At present, some studies have comprehensively investigated the distribution of metal isotopes in plants, including isotopes of Fe^9^,^10^, Zn^11^,^12^,^13^, Cu^14^,^15^, Ca^16^,^17^,^18^,^19^,^20^, Mg^21^,^22^, and Ni^23^. Overall, these studies suggested that the identification of different isotopes within higher plants had specific mode of transport. Hence, metal isotopes could be used as valuable tracers when researching metal uptake, storage and translocation processes within plants.

High precision multiple collector inductively coupled plasma mass spectrometer (MC-ICPMS) has extended the application range of Cd isotopes. Cd stable isotopes were initially used to study mass-dependent fractionation in ordinary chondrites and lunar samples, generated by partial evaporation and condensation^24^,^25^,^26^. In addition, some studies reported that anthropogenic processes might lead to Cd isotopic fractionations, suggesting that Cd stable isotopes could be used as tracers for anthropogenic Cd pollution of the environment^27^,^28^,^29^,^30^. Moreover, many studies have focused on the marine environment, suggesting that biological uptake and utilisation of dissolved seawater Cd generated significant Cd isotope fractionation in the oceans^31^,^32^,^33^,^34^,^35^,^36^,^37^,^38^.

However, to date there has been limited research on Cd isotopic composition in plants. In the present study, three Cd tolerant *Ricinus communis* cultivars (Zibo-5, Zibo-6, Zibo-8) and one Cd hyperaccumulator *Solanum nigrum* cultivar were used to study Cd uptake and translocation. These three *R. communis* cultivars were all high-Cd accumulators, and *S. nigrum* was a relatively fast-growing and high-biomass Cd-hyperaccumulator^39^,^40^ used to develop new techniques for phytoextraction^41^. We conducted hydroponic culture experiments with these plant species and two nutrient solutions with differing Cd concentrations to 1) characterise the Cd isotope fractionation associated with Cd transfer in the Cd-tolerant and -hyperaccumulator species; and 2) explore possible mechanisms of Cd mobilisation from the solution to various physiological compartments.

## Materials and Methods

### Plant Growth

Seeds of three *R. communis* cultivars (Zibo.5, Zibo.6 and Zibo.8) and *S. nigrum* were obtained from the Zibo Academy of Agricultural Sciences (Shandong, China) and the Institute of Applied Ecology, Chinese Academy of Sciences (Shenyang, China), respectively. All seeds were washed in running deionized water before germination in the substrate for 14 d. The seedlings were then transferred into polycarbonate pots containing half strength Hoagland’s solution^39^. The macronutrient solution consisted of 2 mmol·L^−1^ Ca(NO_3_)_2_, 2.5 mmol·L^−1^ KNO_3_, 0.5 mmol·L^−1^ KH_2_PO_4,_ 1 mmol·L^−1^ MgSO_4_ and 0.5 mmol·L^−1^ NH_4_NO_3_, as well as the micronutrient solution consisted of 0.25 μmol·L^−1^ H_3_BO_3_, 0.25 μmol·L^−1^ MnSO_4_, 0.25 nmol·L^−1^ CoCl_2_, 12.5 nmol·L^−1^ KI, 75 nmol·L^−1^ ZnSO_4_, 0.25 nmol·L^−1^ CuSO_4_, 2.5 nmol·L^−1^ Na_2_MoO_4_ and 25 μmol·L^−1^ Fe-EDTA. After 7 d, CdCl_2_·2.5H_2_O was added to the Cd concentration of 2 mg·L^−1^ (Low Cd) and 5 mg·L^−1^ (High Cd). No Cd was added to the control check (CK).

Plants were cultivated under controlled conditions (16 h photoperiod with a white light intensity of 350 μ mol photons m^−2^ s^−1^; day: night temperatures 25 °C: 18 °C; relative humidity 60% ~ 70%). The isotopic composition (δ^114/110^Cd_spex_) of the initial nutrient solution relative to Spex Cd standard solution was +0.14 ± 0.08‰ (2SD, n = 3).

### Sample Preparation

Three plant samples as replicates were harvested 30 d after their transplantation, washed with tap water, and then rinsed thrice with deionized water. Each plant was divided into root, stem and leaf. Plant materials were freeze-dried and weighed prior analysis.

0.2 g of plant samples were digested in concentrated aristar grade HNO_3_ (5 mL) and HF (1 mL) for 48 h in acid-cleaned Teflon beakers. The closed beakers were placed on a hot plate for 8 h at 80 °C and then at 160 °C until the plants were completely digested. Then 2–3 mL of HClO_4_ was added to the digested solutions to remove organic materials. After evaporation at 165–180 °C, the samples were dried and redissolved in 5 mL 1% (v/v) HNO_3_ (to convert the residue into the nitrate form). 2 mL of supernatant were transferred into pre-cleaned polyethylene bottles for the determination of the Cd content. The remaining fractions were evaporated to dryness, redissolved in 10 mol·L^−1^ HCl (to convert the residue into the chloride form), dried again, and finally taken up in 2 mL 2 mol·L^−1^ HCl for loading on columns.

Cd was purified by anionic exchange chromatography from nutrient solution (initial and final), root, stem and leaf following the procedure of Wei *et al*.^42^. Just prior to determination, the solutions were evaporated to near complete dryness and taken up in an appropriate volume of 1% HNO_3_ to obtain the desired Cd concentration for mass spectrometric analysis. The recovery of Cd purification in this study was higher than 95%.

### Cadmium Isotope Analysis

The Cd concentration of nutrient solutions, root, stem and leaf was measured prior to Cd purification by inductively coupled plasma quadrupole mass spectrometry (ICP-QMS) (Elan DRC-e, Perkin Elmer, USA). The Cd isotope ratios were measured by multiple collector inductively coupled plasma mass spectrometry (MC-ICPMS). Cd isotope ratios were measured by 30 cycles for each sample with an internal precision of ±0.01‰ ∼ ±0.02‰ (RSD). The Cd isotope values were expressed as permil deviation relative to the Spex Cd standard solution:


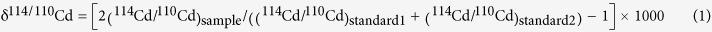


Standard 1 and Standard 2 represented the standard solution measured before and after the sample.

All concentration data were corrected for the procedural blank, which ranged from 8.8 ng to 13.2 ng during the course of this study. At this level, the blank has a negligible effect on the measured isotope compositions, because it constitutes less than 0.0132‰ of the indigenous Cd present in plant samples. Standard-sample bracketing was applied in this study to correct the mass bias. The instrumental reproducibility based on repetitive δ^114/110^Cd measurements of Spex Cd standard solution was 0.09‰ (2SD, N = 214). The accuracy of the measurements was verified by measuring the Münster Cd standard solution and the results (+4.53 ± 0.08‰ of δ^114/110^Cd) were in good agreement with previously published values^30^,^43^.

To express the isotope fractionation between two components A and B, we used δ^114/110^Cd_A−B_ that equaled the difference δ^114/110^Cd_A_ − δ^114/110^Cd_B_.

The isotope composition of the whole plant Cd δ^114/110^ Cd_WP_ has be established according to the following:


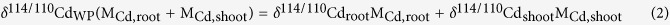


The Cd isotope composition of shoot and the whole plant (WP) relative to Spex Cd standard solution, as well as the isotopic variation between the different organs are shown in Table 2.

### Data Analysis

Cadmium bioconcentration factor (BCF) was defined as the ratio of Cd in shoot or root of the plant to that in the nutrient solution. Cadmium translocation factor (TF) was described as the ratio of Cd in the shoot to that in the root. Tolerance index (TI) was defined as the ratio of the plant biomass after Cd treatments to that of the control group. The indexes were defined as follows:


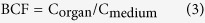


where, C_organ_ (mg·kg^−1^) and C_medium_ (mg·L^−1^) represent the Cd concentration in the shoot or root and the Cd concentration in the nutrient solution, respectively.


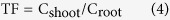


where, C_shoot_ (mg·kg^−1^) and C_root_ (mg·kg^−1^) represent the Cd concentration in the shoot and the Cd concentration in the root, respectively.


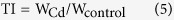


where, W_Cd_ (g) and W_control_ (g) represent the biomass after Cd treatment and the biomass of the control group, respectively.

## Results

### Cd concentration and mass in organs of *R. communis* and *S. nigrum*

**Figure 1:**
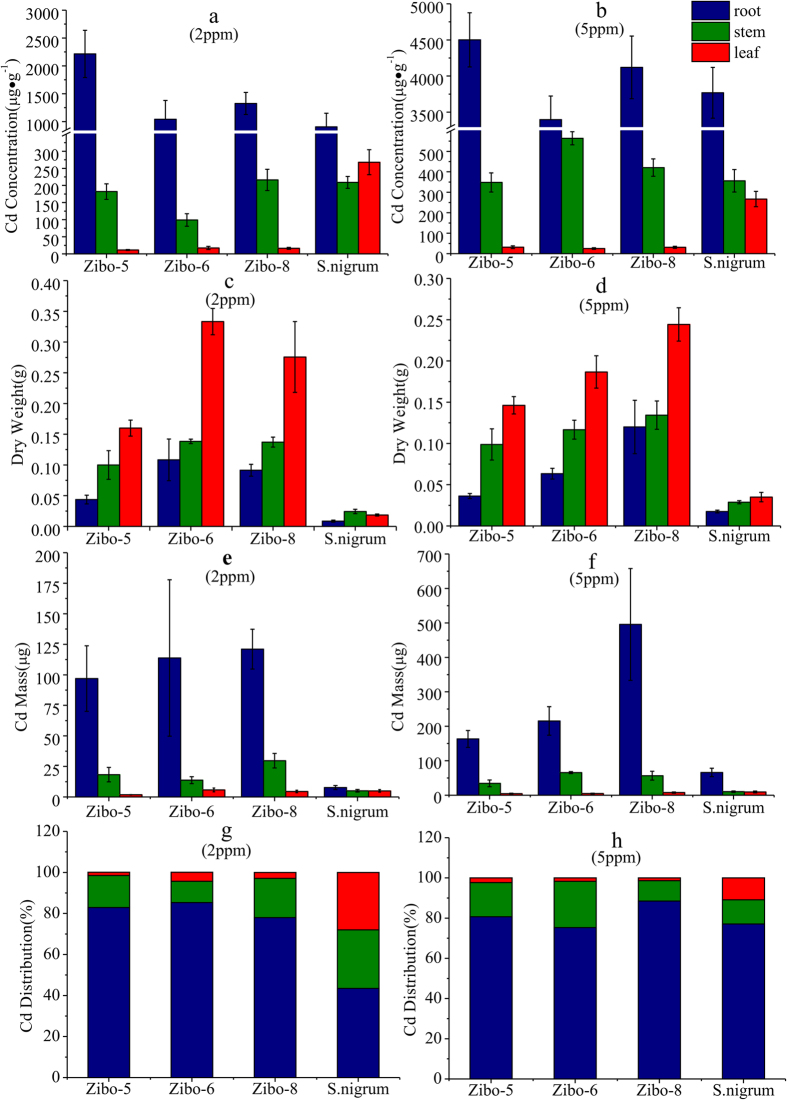
Cd concentration (**a**,**b**), dry weight (**c**,**d**), Cd mass (**e**,**f**) and Cd distribution (**g**,**h**) of root, stem, and leaf of three *R. communis* cultivars and *S. nigrum* during the 2ppm and 5ppm Cd solution conditions. Error bars show standard deviation (SD) of the three replicates.

The Cd concentrations in different organs of *R. communis* and *S. nigrum* are shown in Fig. 1a,b. The Cd concentration of the leaf is much higher in *S. nigrum* than that in *R. communis*, whereas it is equal to or lower in stem and root of *S. nigrum* than that of *R. communis*. Cd concentrations in different organs of *R. communis* exhibit a significant gradient with a progressive increase from upper to lower organs, by the order of leaf <stem <root, independently of the Cd concentration in the nutrient solution. In contrast, the Cd concentration in the leaf of *S. nigrum* is higher than that in the stem under low Cd conditions.

It is essential to precisely determine mass-balances for Cd in the different organs when Cd transfer in the plants is investigated^13^,^44^. The Cd mass is calculated using the dry weight and Cd concentrations of the plant organs as shown in Fig. 1c–f. The total Cd mass in *R. communis* is higher than that in *S. nigrum*. The Cd mass in the root of *R. communis* is higher than that in the shoot independently of low or high Cd conditions. In contrast, Cd mass in the root of *S. nigrum* is much lower than that in the shoot under low Cd conditions. The Cd mass in the two tested plant species exhibits a consistent gradient that progressively increase from upper to lower organs, by the order of leaf <stem< root.

### Cd bioconcentration factor, translocation factor and tolerance index of *R. communis* and *S. nigrum*

All bioconcentration factors (BCFs) of the four plant cultivars are higher than 1 (Table 1). The BCFs of the four cultivars under soil condition are lower than those under hydroponic conditions, considering that Cd in soil occurs in complicated forms because of its association with many physicochemical environments that impact Cd availability. The root BCFs of different cultivars increase by the order of Zibo-5 > Zibo-8 > Zibo-6 > *S. nigrum* under low Cd conditions, whereas they increase by the order of Zibo-5 > Zibo-8 > *S. nigrum* > Zibo-6 under high Cd conditions. The shoot BCFs of different cultivars increase by the order of *S. nigrum* > Zibo-8 > Zibo-5 > Zibo-6 under low Cd conditions, whereas they increase by the order of *S. nigrum* > Zibo-6 > Zibo-8 > Zibo-5 under high Cd conditions. Consequently, the root BCFs are highest in Zibo-5, followed by Zibo-8, whereas the shoot BCFs are highest in *S. nigrum*.

The translocation factors (TFs) of four plant cultivars are low, which indicates that the Cd concentration is higher in root than that in shoot. The TFs of different cultivars increase by the order of *S. nigrum* > Zibo-8 > Zibo-6 > Zibo-5 under low Cd conditions, whereas they increase by the order of *S. nigrum* > Zibo-6 > Zibo-8 > Zibo-5 under high Cd conditions. Thus, *S. nigrum* accumulates the highest Cd concentrations during Cd translocation from root to shoot, whereas Zibo-5 accumulates the least, regardless of Cd concentration in solution.

A tolerance index (TI) based on biomass exposed to heavy metals is used to evaluate the heavy metal toxicity in the plants^45^. The TIs of different cultivars increase by the order of Zibo-6 > Zibo-5 > Zibo-8 > *S. nigrum* under low Cd conditions, whereas they increase by the order of Zibo-5 > Zibo-6 > Zibo-8 > *S. nigrum* under high Cd conditions. *R. communis* reveals higher TI than *S. nigrum* under hydroponic conditions, showing higher Cd tolerance of *R. communis* than *S. nigrum*.

According to an independent samples T-test (p < 0.05), the Cd treatments exert significant effects on transport and accumulation in Zibo-6, Zibo-8, and *S. nigrum* but have no significant effects on shoot accumulation and transport in Zibo-5. Overall, *R. communis* is characterised by a higher Cd tolerance, whereas *S. nigrum* has a higher potential to translocate Cd from root to shoot. In the organs of these four plant cultivars, more Cd is accumulated in the root of *R. communis*, whereas more Cd is translocated from root to shoot in *S. nigrum* than *R. communis*.

### Cd isotopic composition in *R. communis* and *S. nigrum*

**Figure 2:**
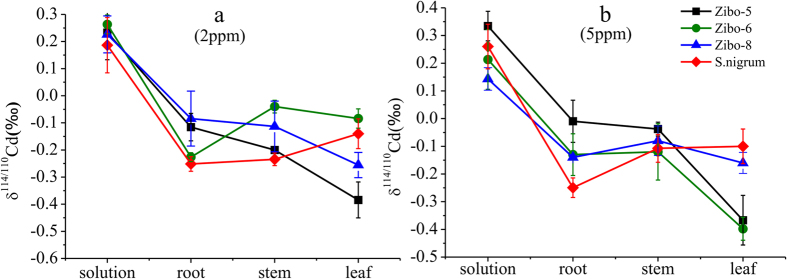
Cd isotope compositions (reported as δ^114/110^Cd_spex_) in final solution, root, stem, and leaf of three *R. communis* cultivars and *S. nigrum*. Error bars show standard deviation (SD) of the three replicates.

The four plant cultivars reveal small differences in δ^114/110^Cd_Stem-Root_ (Table 2). The stem of Zibo-5 is enriched in lighter isotopes relative to the root, whereas Zibo-6 and *S. nigrum* are enriched in heavy isotopes relative to the root. In contrast, the δ^114/110^Cd_Stem-Root_ values of Zibo-8 behave differently under low and high Cd conditions. In low Cd conditions, the stem of Zibo-8 is depleted of heavy isotopes relative to the root, which is consistent with Zibo-5, whereas, in high Cd conditions, the stem of Zibo-8 is enriched in heavy isotopes relative to the root, which is consistent with Zibo-6 and *S. nigrum*. The three *R. communis* cultivars show similar distributions of heavy and light Cd isotopes in stem and leaf, which are different to *S. nigrum*. The leaf of the three *R. communis* cultivars is all enriched in lighter isotopes relative to the stem, whereas those of *S. nigrum* are depleted of light isotopes relative to the stem (Fig. 2).

The Cd isotope compositions in the organs of Zibo-5, Zibo-6, and *S. nigrum* under low and high Cd conditions behave similarly, but differently to Zibo-8. The observed isotopic fractionations between the solution and organs increase by the order of δ^114/110^Cd_Root-Solution_ > δ^114/110^Cd_Stem-Solution_ > δ^114/110^Cd_Leaf-Solution_ for Zibo-5, whereas they increase in the reverse order by δ^114/110^Cd_Leaf-Solution_ >δ^114/110^Cd_Stem -Solution_ >δ^114/110^Cd_Root-Solution_ for *S. nigrum*. In contrast, the isotope value of δ^114/110^Cd_Stem-Solution_ in Zibo-6 is larger than values of δ^114/110^Cd_Root-Solution_ and δ^114/110^Cd_Leaf-Solution_. The Cd isotopic fractionation between the solution and organs of Zibo-8 under low conditions behave similar to Zibo-5, but those under high conditions behave similarly to Zibo-6.

## Discussion

The average δ^114/110^Cd_WP-Solution_ values observed from solution to plants for Zibo-5, Zibo-6, Zibo-8, and *S. nigrum* are −0.36‰, −0.40‰, −0.30‰ and −0.46‰, respectively (Table 2). The observed enrichment of light Cd isotopes is consistent with previous studies on other metal isotopes (e.g. Cu, Fe, Zn, Ca) in plants, except for Mg exhibiting isotopically heavy plant biomass^6^,^13^,^14^,^15^,^23^,^46^. The physiological and molecular mechanisms of Cd hyperaccumulation and tolerance include root proliferation in Cd-rich substrate, influx into cytosol or vacuole by specific and non-specific transporters, and complexation of Cd by certain ligands in cells^47^. Based on the physiological and molecular mechanisms of Cd in higher plants, the speciation and diffusion in solution, adsorption on the root cell walls, uptake by ZIP proteins (Zinc-regulated transporter, iron-regulated transporter protein), complexation by phytosiderophores in solution and uptake of the entire complex through the membrane may affect the metal isotope fractionation^6^,^14^,^44^. The Cd isotopic composition in root and shoot possibly reflects a combination of all these processes.

Two possible abiotic processes could lead to isotope fractionation at the solution-root interface: diffusion and adsorption. Rodushkin *et al*.^48^ found that lighter isotopes diffused faster than heavier isotopes and free ions diffused faster than complex ions. Diffusion from solution to root could lead to an enrichment of the lighter isotopes at the root surface. In addition, adsorption could also result in Cd isotope fractionation. A previous study^49^ showed a small Cd isotope fractionation occurred during sorption of Cd to synthetic birnessite from low ionic strength solution, with lighter isotopes sorbed and heavier isotopes remaining in solution. In the present study, the δ^114/110^Cd _Root-Solution_ in *R. communis* and *S. nigrum* is −0.51‰ to −0.28‰ (Table 2). The root is enriched in the lighter Cd isotope. Therefore, diffusion may be a dominant process, leading to Cd isotope fractionation at the solution-root interface of *R. communis* and *S. nigrum*.

Cd transport across the root cell and other cell membranes are possibly metabolically controlled^14^. In addition, within plants Cd can be transported along the electrochemical gradient via carrier proteins and ion channels or against the electrochemical gradient via electrogenic pumps^13^,^14^. Carrier-mediated transport favours heavy isotopes because it involves covalent binding to a carrier protein on the outer side of the membrane, with subsequent release on the inner side as a result of conformational changes in the carrier^13^. Conversely, transport through ion channels or via electrogenic pumps favours light isotopes because of its greater diffusion coefficient^14^. The observed net enrichment of the lighter isotopes in root and the differences between the plant cultivars, therefore, suggest that membrane transport is dominated by ion channels and electrogenic pumps rather than by carrier-mediated transport.

**Figure 3:**
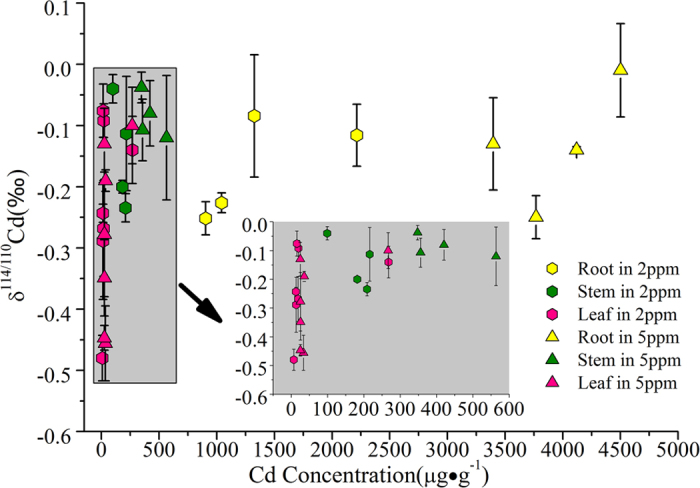
Relationships between the Cd concentration and δ^114/110^Cd in the organs of *R. communis* and *S. nigrum* under different Cd conditions.

The differences in Cd isotopic fractionation from root to stem of four plant cultivars might be due to the different Cd supply limitation, which is associated with the tolerance of plants. Although the Cd mass in nutrient solutions is sufficiently supplied, the Cd mass translocated in the extracellular and cellular plant organs might be limited in different plant cultivars. The magnitudes of the isotopic shifts during the solution-to-organ transfer slightly increase with decreasing Cd concentrations in the organs (Fig. 3). Moreover, the plant biomass is higher under low Cd conditions than that under high Cd conditions (Fig. 1c,d). Therefore, the Cd stress affects the magnitude of the isotopic shift during the solution-to-organ transfer. Gault-Ringold *et al*.^34^ proposed that Cd uptake of phytoplankton did not result in no net Cd isotopic fractionation under ‘supply-limited’ condition, but it could be kinetically driven resulting in Cd isotopic fractionation under sufficiently high Cd levels. This could explain the different Cd isotope fractionation from root to stem between the cultivars.

The variation in the Cd isotopic composition between stem and leaf in *R. communis* and *S. nigrum* is distinct. It may be attributed to the complexation with organic acids, phytochelatins (PCs), and metallothionein in the xylem of *S. nigrum*. Sun *et al*.^50^ identified that complexation with organic acids, phytochelatins (PCs), and metallothionein was an important mechanism for Cd detoxification, transportation and storage in *S. nigrum*. In addition, previous work^14^ also showed that complexation with organic ligands led to an enrichment of heavy isotope in the organs. Figure 1g,h show that a higher amount of Cd is stored in the root of *R. communis*, whereas more Cd is translocated to the stem and leaf of *S. nigrum*. This can be explained by the complexation of organic acids, phytochelatins (PCs), and metallothionein in *S. nigrum* with Cd, which catalyse the translocation of Cd from root to shoot. The observed difference between root and shoot in the Cd-tolerant and -hyperaccumulator species may reflect the different Cd transportation mechanisms of the species.

Until recently, limited studies have reported the Cd isotopic composition in plants, including *Cyperus alternifolius* (−0.37‰ of δ^114/110^Cd_spex_), *Pteris vittata* (−0.34‰ of δ^114/110^Cd_spex_) and some birch leaves (ranged from +0.30‰ to +1.3‰ of δ^114/110^Cd_spex_)^42^,^51^. In the present study, all Cd isotopic compositions of plants determined for *R. communis* (−0.40‰ to −0.01‰) and *S. nigrum* (−0.25‰ to −0.10‰) show negative values relative to the Spex Cd standard solution. This further suggests that these two plant species preferentially take up lighter Cd isotope. In comparison, Pallavicini *et al*.^51^ reported that the δ^114/110^Cd_spex_ values of birch leaves favoured the enrichment of heavier Cd isotopes. Wei *et al*.^42^ suggested that different Cd isotopic compositions in different plant samples could result from distinct mechanisms of Cd accumulation in plants or different sources of Cd (from soil or nutrient solution).

**Figure 4:**
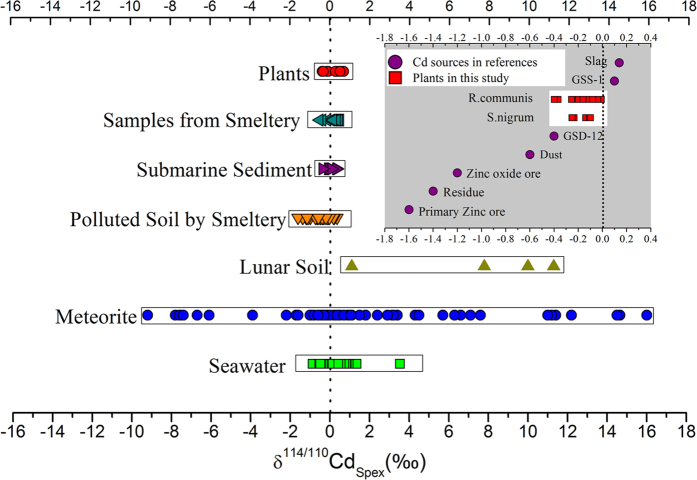
Cd isotopic compositions in plants and other natural materials (reported as δ^114/110^Cd_spex_)^24^,^26^,^28^,^29^,^34^,^35^,^36^,^37^,^38^,^53^,^54^,^55^,^56^,^57^,^58^. GSS-1 and GSD-12 are geological reference materials. GSS-1 is a dark brown podzolitic soil, typical of the mountainous terrain of Northeast China. The underlying granitic bedrock is a part of a lead-zinc mineralisation district. GSD-12 stream sediment is from a tributary draining river in the Yangchun ore field (Cu, W, Sn), Guangdong, China.

The Cd isotopic composition of *R. communis* and *S. nigrum* are enriched with Cd isotope reservoirs in nature. Fig. 4 shows Cd isotope investigations on natural materials, such as meteorites and lunar rocks^24^,^25^,^26^,^52^, seawater^34^,^35^,^36^,^37^,^38^,^53^,^54^,^55^,^56^, samples from Pb-Zn smelting and refining plants^28^,^29^, and soil polluted by the emissions from plants^57^,^58^. Compared with the Cd isotope values in those materials, the variation of Cd isotopic compositions in plants is small. However, plants represent a reservoir of Cd isotopes in nature. In previous studies^28^,^29^,^57^,^58^, the δ^114/110^Cd_spex_ values of source featured with ‘slag (+0.4‰)> GSS-1 (+0.1‰)> GSD-12 (−0.4‰)> dust (−0.6‰)> Zinc oxide ore (−1.2‰)> residue (−1.4‰)> Primary Zinc ore (−1.6‰)’ (Fig. 4). In the present study, the δ^114/110^Cd_spex_ ranges for *R. communis* (−0.40‰ to −0.01‰) and *S. nigrum* (−0.25‰ to −0.10‰) were between the δ^114/110^Cd_spex_ values of GSS-1(soil) and GSD-12 (sediment).

## Conclusions

In the present study, the Cd isotope measurements show an isotopic shift to lighter isotopes during Cd transport from the nutrient solution to the plant organs of the Cd-tolerant *R. communis* and the Cd-hyperaccumulator *S. nigrum*. The observed isotope fractionation is enriched with the Cd isotope reservoirs in nature. In addition, the variation of the Cd isotopic compositions in leaf and stem differs between *R. communis* and *S. nigrum* implying different mechanisms of Cd translocation to the xylem in the Cd-tolerant and -hyperaccumulator species. Cd isotope fractionations of different organs provide new information to identify the chemical processes controlling Cd uptake and translocation in plants and organisms. Plant uptake is an important factor of isotopic variation in the Cd biogeochemical cycle. Thus, Cd isotope fractionation by plants needs to be taken into account in future investigations on environmental pollution using Cd isotopes. Overall, studies on Cd isotopes in plants lay the groundwork for understanding the biogeochemical Cd cycle and mechanisms of plant Cd acquisition and allocation.

## Additional Information

**How to cite this article**: Wei, R. *et al*. Fractionation of Stable Cadmium Isotopes in the Cadmium Tolerant *Ricinus communis* and Hyperaccumulator *Solanum nigrum*. *Sci. Rep*. **6**, 24309; doi: 10.1038/srep24309 (2016).

## Figures and Tables

**Table 1:** Effects of Cd stress on bioconcentration factor (BCF), translocation factor (TF) and tolerance index (TI) of three *R. communis* cultivars and *S. nigrum* in hydroponic conditions.

Plants	Cd treatment	BCF		TF (%)	TI (%)
		root	shoot		
Zibo.5	2ppm	1107.2 ± 211.6a	38.5 ± 8.7a	3.5 ± 0.6 a	104.7 ± 9.4a
	5ppm	900.2 ± 75.0b	31.9 ± 6.7a	3.5 ± 0.7a	97.0 ± 9.1a
Zibo.6	2ppm	521.2 ± 168.3b	20.7 ± 4.6b	4.0 ± 1.3b	141.5 ± 15.0a
	5ppm	679.4 ± 65.3a	46.5 ± 2.8a	6.9 ± 0.8a	89.4 ± 11.8b
Zibo.8	2ppm	662.7 ± 99.3b	41.3 ± 5.7a	6.2 ± 0.1a	84.1 ± 15.8a
	5ppm	824.0 ± 86.8a	33.9 ± 4.7b	4.1 ± 0.9b	83.1 ± 6.0a
*S. nigrum*	2ppm	452.1 ± 123.1b	117.2 ± 11.2a	25.9 ± 8.9a	37.9 ± 9.4b
	5ppm	753.6 ± 70.3a	61.4 ± 8.7b	8.2 ± 1.0b	59.8 ± 11.3a

Mean values (n = 3) with different letters in the same column for each cultivar are significantly different according to the independent samples T-test (p < 0.05).

**Table 2:** δ^114/110^Cd values in root, shoot, and whole plant (WP) of the three *R. communis* cultivars and *S. nigrum* relative to Spex Cd standard solution, as well as the isotopic variations between different organs.

δ^114/110^Cd (‰)	Low Cd (2ppm)				High Cd (5ppm)			
	Zibo-5	Zibo-6	Zibo-8	*S.nigrum*	Zibo-5	Zibo-6	Zibo-8	*S.nigrum*
Root (δ^114/110^Cd_spex_)	−0.12	−0.23	−0.08	−0.25	−0.01	−0.13	−0.14	−0.25
Shoot (δ^114/110^Cd_spex_)	−0.22	−0.05	−0.13	−0.19	−0.08	−0.14	−0.09	−0.10
WP (δ^114/110^Cd_spex_)	−0.14	−0.20	−0.09	−0.22	−0.02	−0.13	−0.13	−0.22
Root-Solution	−0.35	−0.49	−0.31	−0.44	−0.34	−0.34	−0.28	−0.51
Stem-Root	−0.08	0.19	−0.03	0.02	−0.03	0.01	0.06	0.14
Leaf-Stem	−0.18	−0.04	−0.14	0.09	−0.33	−0.28	−0.08	0.01
WP-Solution	−0.37	−0.46	−0.32	−0.41	−0.35	−0.34	−0.27	−0.48
Shoot -WP	−0.08	0.15	−0.04	0.03	−0.05	−0.01	0.04	0.11

## References

[b1] World Health Organization. Exposure to cadmium: a major public health concern, Geneva, Switzerland (2010).

[b2] Sankaran R. P. & Ebbs S. D. Cadmium accumulation in deer tongue grass (*Panicum clandestinum L*.) and potential for trophic transfer to microtine rodents. Environ. Pollut. 148, 580–589 (2007). 17258848 10.1016/j.envpol.2006.11.025

[b3] Staessen J. A. . Environmental exposure to cadmium, forearm bone density, and risk of fractures: prospective population study. The Lancet 353, 1140–1144 (1999). 10.1016/s0140-6736(98)09356-8 10209978

[b4] Ali H., Khan E. & Sajad M. A. Phytoremediation of heavy metals-concepts and applications. Chemosphere 91, 869–881 (2013). 23466085 10.1016/j.chemosphere.2013.01.075

[b5] Huang H. . The phytoremediation potential of bioenergy crop *Ricinus communis* for DDTs and cadmium co-contaminated soil. Bioresour. Technol. 102, 11034–11038 (2011). 21993327 10.1016/j.biortech.2011.09.067

[b6] Wiederhold J. G. Metal stable isotope signatures as tracers in environmental geochemistry. Environ. Sci. Technol. 49, 2606–2624 (2015). 25640608 10.1021/es504683e

[b7] Wojcik M., Sugier P. & Siebielec G. Metal accumulation strategies in plants spontaneously inhabiting Zn-Pb waste deposits. Sci. Total Environ. 487, 313–322 (2014). 24793328 10.1016/j.scitotenv.2014.04.024

[b8] Garcia-Delgado M., Rodriguez-Cruz M. S., Lorenzo L. F., Arienzo M. & Sanchez-Martin M. J. Seasonal and time variability of heavy metal content and of its chemical forms in sewage sludges from different wastewater treatment plants. Sci. Total Environ. 382, 82–92 (2007). 17532025 10.1016/j.scitotenv.2007.04.009

[b9] Kiczka M., Wiederhold J. G., Kraemer S. M., Bourdon B. & Kretzschmar R. Iron isotope fractionation during Fe uptake and translocation in alpine plants. Environ. Sci. Technol. 44, 6144–6150 (2010). 20704211 10.1021/es100863b

[b10] Guelke M. & Von Blanckenburg F. Fractionation of stable iron isotopes in higher plants. Environ. Sci. Technol. 41, 1896–1901 (2007). 17410781 10.1021/es062288j

[b11] Moynier F. . Isotopic fractionation and transport mechanisms of Zn in plants. Chem. Geol. 267, 125–130 (2009).

[b12] Viers J. . Evidence of Zn isotopic fractionation in a soil-plant system of a pristine tropical watershed (Nsimi, Cameroon). Chem. Geol. 239, 124–137 (2007).

[b13] Weiss D. J. . Isotopic discrimination of zinc in higher plants. New Phytol. 165, 703–710 (2005). 15720681 10.1111/j.1469-8137.2004.01307.x

[b14] Jouvin D. . Stable isotopes of Cu and Zn in higher plants: evidence for Cu reduction at the root surface and two conceptual models for isotopic fractionation processes. Environ. Sci. Technol. 46, 2652–2660 (2012). 22296233 10.1021/es202587m

[b15] Weinstein C. . Isotopic fractionation of Cu in plants. Chem. Geol. 286, 266–271 (2011).

[b16] Schmitt A. D. . Calcium isotope fractionation during plant growth under a limited nutrient supply. Geochim. Cosmochim. Acta 110, 70–83 (2013).

[b17] Hindshaw R. S. . Calcium isotope fractionation in alpine plants. Biogeochemistry 112, 373–388 (2012).

[b18] Cobert F. . Experimental identification of Ca isotopic fractionations in higher plants. Geochim. Cosmochim. Acta 75, 5467–5482 (2011).

[b19] Cenki-Tok B. . The impact of water-rock interaction and vegetation on calcium isotope fractionation in soil- and stream waters of a small, forested catchment (the Strengbach case). Geochim. Cosmochim. Acta 73, 2215–2228 (2009).

[b20] von Blanckenburg F., von Wiren N., Guelke M., Weiss D. J. & Bullen T. D. Fractionation of metal stable isotopes by higher plants. Elements 5, 375–380 (2009).

[b21] Bolou-Bi E. B., Poszwa A., Leyval C. & Vigier N. Experimental determination of magnesium isotope fractionation during higher plant growth. Geochim. Cosmochim. Acta 74, 2523–2537 (2010).

[b22] Black J. R., Epstein E., Rains W. D., Yin Q. Z. & Casey W. H. Magnesium-isotope fractionation during plant growth. Environ. Sci. Technol. 42, 7831–7836 (2008). 19031868 10.1021/es8012722

[b23] Deng T. H. . Nickel and zinc isotope fractionation in hyperaccumulating and nonaccumulating plants. Environ. Sci. Technol. 48, 11926–11933 (2014). 25222693 10.1021/es5020955

[b24] Wombacher F., Rehkamper M., Mezger K., Bischoff A. & Munker C. Cadmium stable isotope cosmochemistry. Geochim. Cosmochim. Acta 72, 646–667 (2008).

[b25] Schediwy S., Rosman K. J. R. & de Laeter J. R. Isotope fractionation of cadmium in lunar material. Earth Planet. Sci. Lett. 243, 326–335 (2006).

[b26] Sands D. G., Rosman K. J. R. & de Laeter J. R. A preliminary study of cadmium mass fractionation in lunar soils. Earth Planet. Sci. Lett. 186, 103–111 (2001).

[b27] Chrastný V. . Cadmium isotope fractionation within the soil profile complicates source identification in relation to Pb-Zn mining and smelting processes. Chem. Geol. 405, 1–9 (2015).

[b28] Shiel A. E., Weis D. & Orians K. J. Evaluation of zinc, cadmium and lead isotope fractionation during smelting and refining. Sci. Total Environ. 408, 2357–2368 (2010). 20206962 10.1016/j.scitotenv.2010.02.016

[b29] Cloquet C., Carignan J., Libourel G., Sterckeman T. & Perdrix E. Tracing source pollution in soils using cadmium and lead isotopes. Environ. Sci. Technol. 40, 2525–2530 (2006). 16683587 10.1021/es052232+

[b30] Gao B. . Precise determination of cadmium and lead isotopic compositions in river sediments. Anal. Chim. Acta 612, 114–120 (2008). 18331865 10.1016/j.aca.2008.02.020

[b31] Conway T. M. & John S. G. Biogeochemical cycling of cadmium isotopes along a high-resolution section through the North Atlantic Ocean. Geochim. Cosmochim. Acta 148, 269–283 (2015).

[b32] Georgiev S. V. . Cadmium isotopic evidence for increasing primary productivity during the Late Permian anoxic event. Earth Planet. Sci. Lett. 410, 84–96 (2015).

[b33] Horner T. J., Lee R. B., Henderson G. M. & Rickaby R. E. Nonspecific uptake and homeostasis drive the oceanic cadmium cycle. Proc. Natl. Acad. Sci. USA 110, 2500–2505 (2013). 23362377 10.1073/pnas.1213857110 PMC3574901

[b34] Gault-Ringold M., Adu T., Stirling C. H., Frew R. D. & Hunter K. A. Anomalous biogeochemical behavior of cadmium in subantarctic surface waters: mechanistic constraints from cadmium isotopes. Earth Planet. Sci. Lett. 341–344, 94–103 (2012).

[b35] Abouchami W. . Modulation of the Southern Ocean cadmium isotope signature by ocean circulation and primary productivity. Earth Planet. Sci. Lett. 305, 83–91 (2011).

[b36] Schmitt A. D., Galer S. J. G. & Abouchami W. High-precision cadmium stable isotope measurements by double spike thermal ionisation mass spectrometry. J. Anal. Atom. Spectrom. 24, 1079–1088 (2009).

[b37] Ripperger S. & Rehkamper M. Precise determination of cadmium isotope fractionation in seawater by double spike MC-ICPMS. Geochim. Cosmochim. Acta 71, 631–642 (2007).

[b38] Lacan F., Francois R., Ji Y. C. & Sherrell R. M. Cadmium isotopic composition in the ocean. Geochim. Cosmochim. Acta 70, 5104–5118 (2006).

[b39] Zhang H. . Cadmium accumulation and tolerance of two castor cultivars in relation to antioxidant systems. J. Environ. Sci. (China) 26, 2048–2055 (2014). 25288549 10.1016/j.jes.2014.08.005

[b40] Wei S. H., Zhou Q. X. & Koval P. V. Flowering stage characteristics of cadmium hyperaccumulator *Solanum nigrum L*. and their significance to phytoremediation. Sci. Total Environ. 369, 441–446 (2006). 16859734 10.1016/j.scitotenv.2006.06.014

[b41] Chen L. . Interaction of Cd-hyperaccumulator *Solanum nigrum L*. and functional endophyte Pseudomonas sp Lk9 on soil heavy metals uptake. Soil Biol. Biochem. 68, 300–308 (2014).

[b42] Wei R. . An analytical method for precise determination of the cadmium isotopic composition in plant samples using multiple collector inductively coupled plasma mass spectrometry. Anal. Methods 7, 2479–2487 (2015).

[b43] Abouchami W. . A common reference material for cadmium isotope ctudies-NIST SRM 3108. Geostandard. Geoanal. Res. 37, 5–17 (2013).

[b44] Aucour A. M., Pichat S., Macnair M. R. & Oger P. Fractionation of stable zinc isotopes in the zinc hyperaccumulator *Arabidopsis halleri* and nonaccumulator *Arabidopsis petraea*. Environ. Sci. Technol. 45, 9212–9217 (2011). 21882835 10.1021/es200874x

[b45] Shi G. & Cai Q. Cadmium tolerance and accumulation in eight potential energy crops. Biotechnol. Adv. 27, 555–561 (2009). 19393309 10.1016/j.biotechadv.2009.04.006

[b46] Tang Y. T. . Fractionation of stable zinc isotopes in the field-grown zinc hyperaccumulator *Noccaea caerulescens* and the zinc-tolerant plant *Silene vulgaris*. Environ. Sci. Technol. 46, 9972–9979 (2012). 22891730 10.1021/es3015056

[b47] Qiu R. L. . Mechanisms of Cd hyperaccumulation and detoxification in heavy metal hyperaccumulators: How Plants Cope with Cd. 73, 127–159 (2012).

[b48] Rodushkin I., Stenberg A., Andren H., Malinovsky D. & Baxter D. C. Isotopic fractionation during diffusion of transition metal ions in solution. Anal. Chem. 76, 2148–2151 (2004). 15053683 10.1021/ac035296g

[b49] Wasylenki L. E., Swihart J. W. & Romaniello S. J. Cadmium isotope fractionation during adsorption to Mn oxyhydroxide at low and high ionic strength. Geochim. Cosmochim. Acta 140, 212–226 (2014).

[b50] Sun R. L., Zhou Q. X., Sun F. H. & Jin C. X. Antioxidative defense and proline/phytochelatin accumulation in a newly discovered Cd-hyperaccumulator, Solanum nigrum L. Environ. Exp. Bot. 60, 468–476 (2007).

[b51] Pallavicini N. . Cadmium isotope ratio measurements in environmental matrices by MC-ICP-MS. J. Anal. Atom. Spectrom. 29, 1570–1584 (2014).

[b52] Wombacher F., Rehkamper M., Mezger K. & Munker C. Stable isotope compositions of cadmium in geological materials and meteorites determined by multiple-collector ICPMS. Geochim. Cosmochim. Acta 67, 4639–4654 (2003).

[b53] Lambelet M. . Isotopic analysis of Cd in the mixing zone of Siberian rivers with the Arctic Ocean-new constraints on marine Cd cycling and the isotope composition of riverine Cd. Earth Planet. Sci. Lett. 361, 64–73 (2013).

[b54] Xue Z., Rehkamper M., Schonbachler M., Statham P. J. & Coles B. J. A new methodology for precise cadmium isotope analyses of seawater. Anal. Bioanal. Chem. 402, 883–893 (2012). 22033821 10.1007/s00216-011-5487-0

[b55] Yang S. C., Lee D. C. & Ho T. Y. The isotopic composition of cadmium in the water column of the South China Sea. Geochim. Cosmochim. Acta 98, 66–77 (2012).

[b56] Ripperger S., Rehkamper M., Porcelli D. & Halliday A. N. Cadmium isotope fractionation in seawater-a signature of biological activity. Earth Planet. Sci. Lett. 261, 670–684 (2007).

[b57] Wen H. . Tracing sources of pollution in soils from the Jinding Pb-Zn mining district in China using cadmium and lead isotopes. Appl. Geochem. 52, 147–154 (2015).

[b58] Gao B., Zhou H. D., Liang X. R. & Tu X. L. Cd isotopes as a potential source tracer of metal pollution in river sediments. Environ. Pollut. 181, 340–343 (2013). 23809663 10.1016/j.envpol.2013.05.048

